# Experimental Everglades Virus Infection of Cotton Rats (*Sigmodon hispidus*)

**DOI:** 10.3201/eid1012.040442

**Published:** 2004-12

**Authors:** Lark L. Coffey, Anne-Sophie Carrara, Slobodan Paessler, Michelle L. Haynie, Robert D. Bradley, Robert B. Tesh, Scott C. Weaver

**Affiliations:** * University of Texas Medical Branch, Galveston, Texas, USA;; † Texas Tech University, Lubbock, Texas, USA

**Keywords:** Everglades virus, cotton rat, Venezuelan equine encephalitis, experimental infection, ecology, research

## Abstract

Everglades virus (EVEV), an alphavirus in the Venezuelan equine encephalitis (VEE) serocomplex, circulates among rodents and vector mosquitoes and infects humans, causing a febrile disease sometimes accompanied by neurologic manifestations. EVEV circulates near metropolitan Miami, which indicates the potential for substantial human disease, should outbreaks arise. We characterized EVEV infection of cotton rats in South Florida, USA, to validate their role in enzootic transmission. To evaluate whether the viremia induced in cotton rat populations regulates EVEV distribution, we also infected rats from a non–EVEV-endemic area. Viremia levels developed in rats from both localities that exceeded the threshold for infection of the vector. Most animals survived infection with no signs of illness, despite virus invasion of the brain and the development of mild encephalitis. Understanding the mechanisms by which EVEV-infected cotton rats resist clinical disease may be useful in developing VEE therapeutics for equines and humans.

## Abstract 2

We characterized Everglades virus infection of cotton rats from South Florida to validate their role as reservoir hosts in the enzootic transmission cycle.

Everglades virus (EVEV; *Togaviridae*: *Alphavirus*) circulates among rodents and vector mosquitoes in South Florida and can tangentially infect humans, causing a febrile disease with occasional neurologic signs. The most closely related Venezuelan equine encephalitis (VEE) complex viruses, enzootic subtype ID strains, are the progenitors of subtype IAB and IC strains responsible for major epidemics and epizootics (*1*). This relationship raises the possibility of epidemic emergence in South Florida, involving mutations in the EVEV genome, with serious public health consequences for >2 million people in metropolitan Miami-Dade County.

EVEV was first recognized in South Florida in the 1960s, when Seminole persons living north of Everglades National Park were shown to have seroprevalence as high as 58% (*2*). Recorded EVEV activity has been limited to South-Central Florida from Everglades National Park, north to Indian River County (Figure 1) (*3**–**10*). Although EVEV circulation in South Florida has been documented repeatedly, little is known about the dynamics of its ecology and transmission. Strains isolated from mosquitoes, laboratory transmission experiments (*11*), and rodent host preferences (*12*) found that *Culex* (*Melanoconion*) *cedecei* was the primary vector. Field studies in the 1960s implicated cotton rats (*Sigmodon hispidus*, subspecies not reported) and cotton mice (*Peromyscus gossypinus*) as reservoirs on the basis of high seroprevalence for EVEV (*4**–**6**,**8*). Subsequent studies characterized EVEV and related VEEV infection in experimentally infected cotton rats and laboratory rodents (*13**–**16*). However, the duration and magnitude of viremia titers needed to infect mosquito vectors, the clinical outcome of infection, and the immune response were never defined in cotton rats from the enzootic region. To better understand the enzootic EVEV cycle, we experimentally infected cotton rats from South Florida.

**Figure 1:**
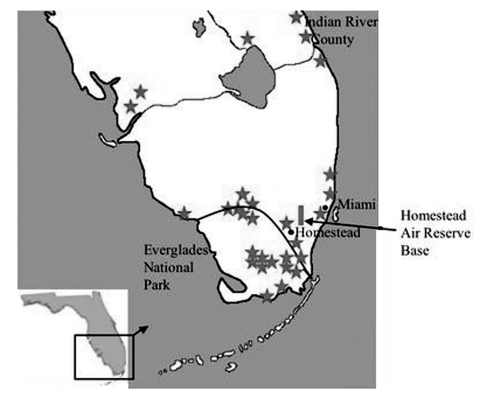
Map of South Florida, indicating locations of Everglades virus isolation, human cases or antibody detection (stars), and our cotton rat collection site (box). Dark line delineates national park boundary.

Factors that regulate the geographic distributions of arboviruses are poorly understood. Animals of different genetic backgrounds can show differential susceptibility and responses to infection with mosquito- and rodentborne viruses (*17**–**19*). For example, rodents most closely related to reservoir species of hantaviruses are more susceptible to infection than more distantly related species are (*20*). Variation in the susceptibility of cotton rats to EVEV might explain why its distribution is restricted to South Florida. Twelve currently recognized subspecies of cotton rats native to the southern United States (*21*) differ by as much as 5% in their cytochrome b DNA sequences (*22*). If genetically distinct cotton rat populations living outside the EVEV-enzootic region do not sustain the magnitude or duration of viremia titers needed to infect sufficient numbers of vectors, they could be incapable of maintaining virus circulation. To test this hypothesis, we compared EVEV infection in a sympatric cotton rat subspecies to infection in a genetically divergent Texas subspecies outside the known EVEV and VEE complex alphavirus distribution (*22*).

## Materials and Methods

### Virus Strains

Two EVEV strains from ENP were used in the experimental infections: the prototype strain, FE3-7c, isolated in 1963 from *Culex* (*Melanoconion*) spp. mosquitoes, was passaged five times in suckling mouse brains (SMB) and twice in Vero cells (*23*), and FE4-71k (SMB1, Vero 1), a 1964 isolate from *Culex* spp. mosquitoes. Both isolates were used to assess strain variation and to determine any effects of the more extensive passage history of FE3-7c on infection or virulence. Virus stocks were prepared in Vero cells, and each animal was inoculated with approximately 1,000 PFU. All inocula were back-titered by plaque assay to determine the exact dose administered.

### Cotton Rat Collection, Identification, and Colonization

Cotton rats were collected in baited live traps (Sherman Traps Inc., Tallahassee, FL) in April 2003 in Homestead Air Reserve Base (25.49°N, 80.38°W) within the EVEV-enzootic region of southern Florida. All procedures were approved by the University of Texas Medical Branch Institutional Animal Care and Use Committee and were performed in accordance with published guidelines (*24*). All rats were seronegative and virus-negative for several rodentborne pathogens enzootic in South Florida, including hantaviruses, arenaviruses, eastern equine encephalitis virus, and EVEV. First generation (F_1_) offspring from mating pairs established in the laboratory were used for infections. To represent a cross-section of the natural population, rats of various ages (3, 6, 9–12 weeks) were infected. In most cases, infected animals and mock-infected controls were matched for age and sex. In addition to morphologic identification of the animals to the species level, DNA was extracted from the liver and purified by using the DNeasy extraction kit (Qiagen, Valencia, CA) or whole blood using the methods of Longmire et al. (*25*), and the cytochrome b gene was amplified and sequenced as described previously for cotton rat identification (*22**,**26*).

A second cohort of cotton rats, representing a different subspecies, was collected in Galveston Island State Park (29.27°N, 94.83°W) in June and August 2003 and used directly for experimental infections. Texas rats were chosen for the following reasons: 1) among U.S. subspecies, Texas cotton rats are the most divergent genetically from the Florida subspecies (*26*) and may exhibit a difference in susceptibility; 2) because the subspecies of cotton rats in which EVEV activity was detected previously is unknown, we wanted to test a subspecies unexposed to VEEV complex viruses; and 3) the use of local rats simplified animal use protocols. Although no VEE complex alphaviruses are known to circulate in Texas, all rats were tested and determined to be EVEV seronegative before infection. The ages of the field-collected rats were unknown, but their weights ranged from 50 g to 160 g, which represents the range of ages in natural populations of cotton rats because weight can be used to estimate life stage and age (*27*). The Texas rats were matched for sex and size, and the cytochrome b gene was sequenced.

### Cotton Rat Infections

Cohorts of eight cotton rats from each location were injected subcutaneously (SC) in the left thigh with EVEV, and two rats per cohort were sham-injected with diluent. The virus dose (2.3–3.6 log_10_ PFU) and infection route is an appropriate simulation of the bite of alphavirus-infected mosquitoes (*28**,**29*). Individually housed animals were monitored daily for signs of illness typical of VEE complex virus infection and were bled from the retroorbitus at 1- to 2-day intervals, beginning 1 day postinfection.

In a subsequent experiment, 15 Florida rats (5–22 weeks of age) were administered 3.2 log_10_ PFU of strain FE4-71k SC and were serially killed at daily intervals (two rats/day) for histologic examination and virus assay of selected organs. Surviving animals were bled daily. Anesthetized rats were perfused with 20 mL to 50 mL of phosphate-buffered saline to eliminate viremic blood from the organs, and organs were homogenized (MM300 homogenizer, Retsch Inc., Newton, PA) in Eagle's minimum essential medium (MEM) with 5% fetal bovine serum to yield a 10% weight/volume suspension. Each suspension was centrifuged at 5,760 x *g* for 6 min, and the supernatant was frozen at –80°C. Additional tissue samples were transferred to 10% formalin for 48 h and then stored in 70% ethanol before being embedded in paraffin, sectioned, and stained with hematoxylin and eosin. Sections were examined in a blinded manner for histopathologic lesions characteristic of VEEV infections of mice and hamsters (*30**,**31*).

### Virus and Antibody Assays

Serum and organ samples were tested for EVEV by plaque assay on Vero cells (*32*). Log- transformed viremia levels were compared among cohorts by using the Mann-Whitney U test (*33*). The limit of detection of the assay was 80 PFU/mL (1.9 log_10_ PFU/mL). Antibody titers were measured by standard 80% plaque reduction neutralization tests (PRNT) (*32*).

## Results

### Identification of Cotton Rats

Genetic distances among mitochondrial cytochrome b gene sequences of rats from Florida and Texas were obtained by using the Kimura 2-parameter model (*34*) and were used to construct a neighbor-joining tree (*35*) that reflected phylogenetic relationships (data not shown). Texas rats grouped closely with *S. hispidus berlandieri*, and Florida rats were identified as *S. hispidus spadicipygus*, another subspecies that differs by up to 5% in its sequence from *berlandieri*, which suggests that these populations represent the maximum level of divergence within the United States.

### Infection Profile and Virus Replication Kinetics

**Figure 2:**
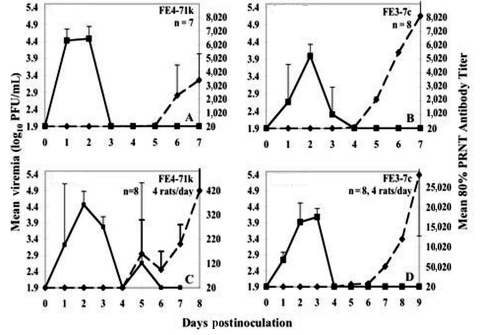
Viremia and neutralizing antibody profile in F_1_ Florida (panels A,B) or wild Texas cotton rats (panels C,D) injected with Everglades virus strains FE4-71k (A, C) and FE3-7c (B, D) administered subcutaneously in the left thigh. Inoculum doses were as follows: panels A and B: 2.9 log_10_ PFU/mL, panel C: 2.3 log_10_ PFU/mL, panel D: 3.6 log_10_ PFU/mL. Florida animals were bled daily; viremia or 80% plaque reduction neutralization test (PRNT) antibody titers represent geometric means of data from eight rats (strain FE3-7c) or seven rats (EVEV FE4-71k). Rats from Texas were each bled every 2 days; means (geometric) represent measurements from four animals. Bars denote standard deviations.

A total of 46 of the 47 cotton rats from both localities injected with EVEV became viremic for 3 to 4 days (Figure 2). With the exception of a single death approximately 30 hours postinfection, all rats survived, and none exhibited detectible illness. The rat that died had viremia and organ titer levels comparable to levels in other rats 1 day postinfection. Rats from Texas did not experience viremia levels of shorter duration or lower magnitude (p > 0.05) than Florida animals, causing us to reject our hypothesis that Texas animals are less likely to exhibit EVEV viremia. Strain FE3-7c produced lower viremia titers than FE4-71k at 1 and 2 days postinfection; however, only differences in Florida rats were significant (p = 0.02 day 1, p = 0.03, day 2). Mean peak titers occurred 2–3 days postinfection and reached 4–4.5 log_10_ PFU/mL for all cohorts. By day 4, viremia levels were not detectable in most rats. Viremia profiles were independent of sex, age, or sibling relatedness among the colony Florida rats (data not shown).

### Pathologic Manifestations and Viral Tropism

**Figure 3:**
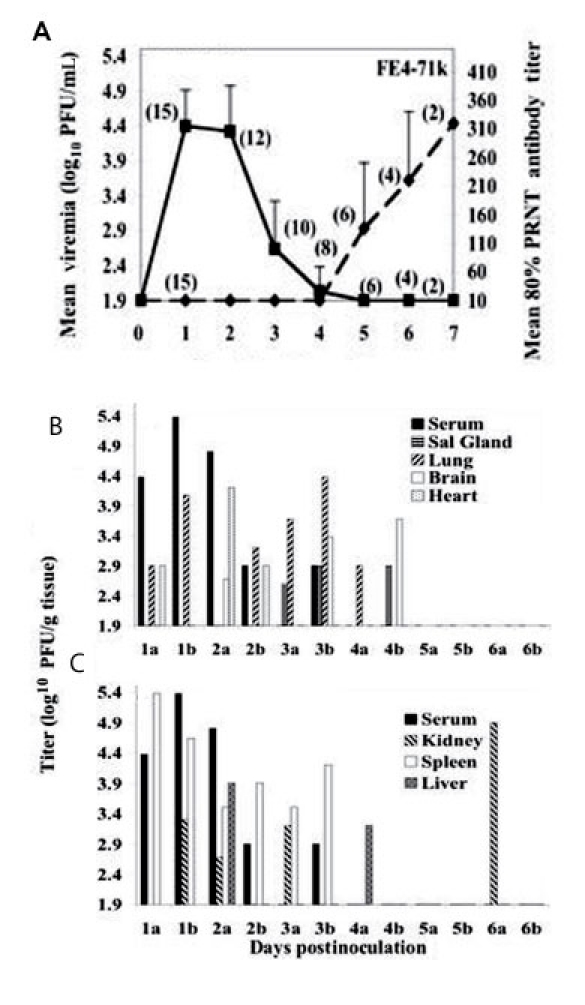
A) Viremia and neutralizing antibody profiles in F_1_ Florida cotton rats serially sacrificed at daily intervals after infection with 3.2 log_10_ PFU of Everglades virus strain FE4-71k administered subcutaneously in the left thigh. Lines on each graph represent the geometric mean viremia or mean 80% plaque reduction neutralization test (PRNT) antibody titers; the number of rats bled at each time point is denoted in parentheses above each point. Error bars denote standard deviations. Everglades virus organ titers from the brain, salivary glands, lung and heart (B) and liver, kidney, and spleen (C) of EVEV strain FE471k-infected F_1_ Florida cotton rats serially sacrificed at daily intervals. Two rats, denoted "a" and "b" were sacrificed daily from days 1–7 postinfection. No virus was detected in any organ on day 7.

Although a single rat died approximately 30 hours postinfection, none of the other 46 infected rats exhibited signs of illness. The viremia profile for rats sacrificed daily (Figure 3, panel A) showed no difference from that generated in the first experiment with animals from the same location infected with the same virus isolate (Figure 2, panel A). Figure 3 shows the temporal course of organ infection in the heart, brain, salivary glands, and lungs (B) and spleen, kidney and liver (C). EVEV was detected in the heart (1–2 days postinfection), salivary glands (3–4 days postinfection), lungs (1–4 days postinfection), brain (2–4 days postinfection) (Figure 3, panel B), and in the spleen (1–3 days postinfection), and inconsistently in the liver (2–4 days postinfection) and kidney (1–6 days postinfection) (Figure 3, panel C). Aside from virus in the kidney of one rat at day 6 postinfection (Figure 3, panel C), virus was cleared from all organs by day 5, which coincided with the development of neutralizing antibodies (described below). We were unable to detect virus in urine or fecal samples collected 1–7 days postinfection.

**Figure 4:**
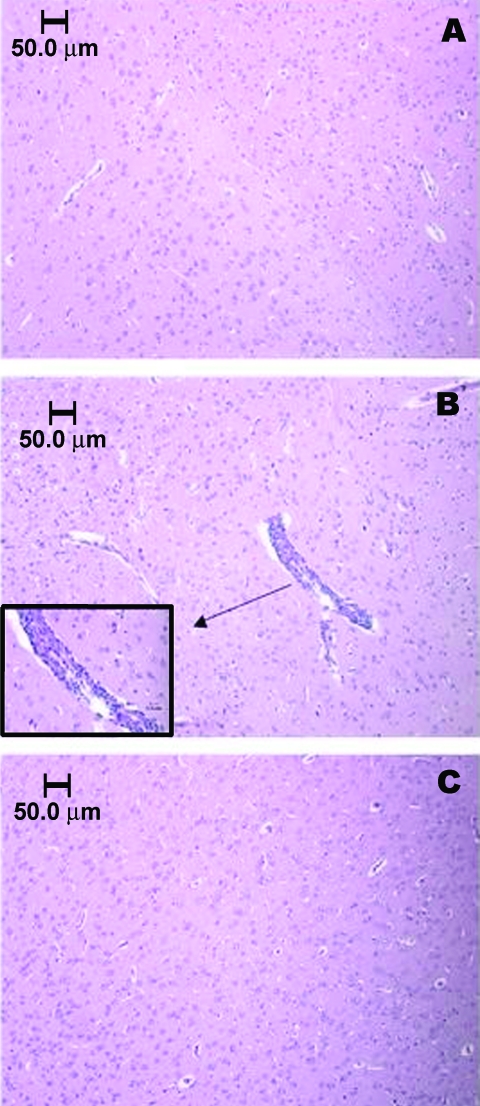
Brainstem section of sham-inoculated control rat, showing the absence of an inflammatory response (A). Vascular and perivascular infiltration of mononuclear cells within the brainstem of a Florida cotton rat 7 days after infection with 3.2 log_10_ PFU/mL EVEV strain FE4-71k; inset enlarged to show cell infiltration (B). Cortex of cotton rat 5 weeks after infection, showing absence of inflammatory response (C). Animals in panels A and B were anesthetized with pentobarbital and perfused with phosphate-buffered saline intracardially. The rat in panel C was not perfused.

Histopathologic examinations showed depletion of lymphoid cells in the spleen on day 2, followed by architectural reorganization and recovery 3 to 7 days postinfection (not shown). Brains of infected rats appeared similar to those of mock-infected rats until 4 days postinfection, coincident with virus clearance from the blood. After day 4, focal meningoencephalitis and associated perivascular mononuclear cell infiltration and neurophagia were observed. Figure 4 shows brain sections from sham-inoculated (A) and encephalitic rats infected with strain FE4-71k that were killed on day 7 postinfection (B), and approximately 5 weeks postinfection (C). The focal encephalitis observed in infected rats at 7 days postinfection was resolved by 5 weeks postinfection, without chronic inflammation or tissue reorganization.

### Antibody Responses

All 46 surviving cotton rats seroconverted; neutralizing antibody was first detectable 5 days postinfection, concordant with or after the disappearance of viremia (Figure 2, panels A–D, Figure 3, panel A). Levels of neutralizing antibody rose rapidly to maximum mean titers of 320 to 28,157 (Figure 2, panel A–D, Figure 3, panel A), and some rats maintained high (>10,240) neutralizing antibody titers for 6 months.

## Discussion

### Infection Outcome

EVEV produced benign, systemic infection when delivered SC in relevant doses to cotton rats from EVEV-endemic and EVEV-nonendemic areas of the United States, and all surviving animals seroconverted. The appearance of antibody sometimes followed the disappearance of viremia, indicating that innate immune mechanisms may participate in virus clearance or that undetectable levels of neutralizing antibody may have preceded the disappearance of viremia. The lower levels of viremia generated by strain FE3-7c may reflect the more extensive cell culture passage history of this isolate. The nonfatal outcome of infection, combined with the high levels of viremia and observations from field studies (*4**–**6**,**8*), is consistent with the role of cotton rats as reservoir hosts for EVEV.

EVEV was neuroinvasive in cotton rats and caused transient, focal encephalitis as well as mild viscerotropic diseases, similar to those caused by other VEE complex alphaviruses. Although encephalitis developed in cotton rats, their ability to clear virus from the brain and the relatively minor inflammatory response they mounted contrasts dramatically with EVEV or VEEV infection of mice (*36*) and warrants further study.

In many respects, our results were similar to published cotton rat infection profiles of animals and VEE complex viruses from other localities. Wild-caught Panamanian cotton rats (probably *S. h. hirsutus*) (*26*) that had been injected with 2.8 log_10_ PFU of an enzootic VEEV subtype ID strain exhibited no virus-induced deaths, but viremia titers developed of 3.7 days mean duration with a peak median magnitude of 7.1 log_10_ Vero PFU/mL at day 2 postinfection (*13*), three orders of magnitude higher than the viremia levels we measured. Howard (*16*) reported that 9 (45%) of 20 cotton rats captured in central Florida near Tampa died after injection with 3.8 log_10_ suckling mouse intracerebral lethal dose 50% (SCILD_50_) of a VEEV subtype IAB isolate, and a peak viremia level of 6.0 log_10_ PFU/mL developed in the surviving animals at day 2 postinfection. Possible explanations for the differences in VEE complex viremia levels in different rat populations include the following: 1) EVEV may generally replicate at lower levels in a variety of rodents, or 2) cotton rats from southern Florida are more resistant to the replication of VEE complex alphaviruses. Infection of cotton rats from southern Florida with other VEE complex strains is needed to test this hypothesis.

The only other reported experimental infections of North American cotton rats with EVEV involved seven animals from Homestead, Florida (C. Calisher, pers. comm.), which became viremic 2–4 days postinfection, with a peak of 6.4 log_10_ SMICLD_50_/mL 3 days postinfection and no deaths (*15*). This peak viremia level is approximately equal to our 4.0 PFU/mL value measured by plaque assays, since the SMICLD_50_:PFU ratio for EVEV is approximately 200:1 (L. L. Coffey, unpub. data).

### Cotton Rats as Reservoirs of EVEV

The fact that high numbers of infected cotton rats in our study survived contrasts with results from EVEV infections of laboratory rodents and is consistent with their role as natural reservoirs. Golden Syrian hamsters and Swiss albino mice experience 75%–100% mortality with doses as low as 3 log_10_ Vero PFU, and pathologic lesions develop, consistent with VEE-like disease (*14**,**30**,**37**,**38*). Even though infection of laboratory rodents often causes death, EVEV infection is less virulent than most other VEE complex viruses, which generally cause 100% of infected animals to die (*14**,**30**,**31**,**38*).

For EVEV transmission by a vector, the reservoir must attain a threshold viremia level (minimum virus titer that infects approximately 1%–5% of vectors [*39*]). Susceptibility studies of *Culex* (*Mel*.) *cedecei* indicated that hamster blood meal titers as low as 0.9 log_10_ chicken embryo cell (CEC) PFU/mL (even lower than the viremia detection limit in our study) infected 9% of mosquitoes, and infected *Cx. cedecei* transmitted EVEV to naïve animals after extrinsic incubation (*11*). With oral doses of 4.9 log_10_ CEC PFU/mL, slightly higher than the peak viremia levels observed in our cotton rats, 100% of *Cx. cedecei* became infected. One EVEV Vero cell PFU approximates one CEC PFU (L. L. Coffey, unpub. data), indicating that the infection threshold for *Cx. cedecei* is lower than the detection limits of our assays. Therefore, any viremia levels we observed should be sufficient to infect at least some *Cx. cedecei*.

The absence of virus in excreta from any of the infected animals indicates that EVEV is probably not transmitted horizontally between nest-mates through this route, despite the detection of virus in the kidney. However, the possibility of persistent infection should be addressed in further studies.

### EVEV Distribution

Our data do not support the hypothesis that variation in the susceptibility of cotton rats explains the limited EVEV distribution. Another explanation supported by susceptibility testing (*11*) is that the mosquito vector limits EVEV distribution. The recorded distribution of *Cx. cedecei* is restricted to 13 counties in South Florida (*40*) and closely parallels the recorded distribution of EVEV activity.

### Potential for EVEV Disease

Understanding arbovirus transmission cycles is important for delineating the epidemiology of human disease. Our data support the role of cotton rats as EVEV reservoirs in South Florida. Future work should focus on cotton rat ecology, with emphasis on population dynamics. Combined with quantitative information about vector-reservoir contact, mosquito population fluctuations, and virus circulation intensities, EVEV transmission dynamics can be elucidated.

Previous studies (*1**,**41*) indicate that epidemic VEEV emerges from enzootic subtype ID strains, the closest relatives of EVEV. Only a few mutations in enzootic VEEV can generate viruses with equine amplification phenotypes (*42*). If such epidemic EVEV strains arise, substantial human illness or deaths could occur. Reverse genetic studies under way in our laboratory are designed to assess this possibility.
